# Validation of biomarkers to predict response to immunotherapy in cancer: Volume II — clinical validation and regulatory considerations

**DOI:** 10.1186/s40425-016-0179-0

**Published:** 2016-11-15

**Authors:** Kevin K. Dobbin, Alessandra Cesano, John Alvarez, Rachael Hawtin, Sylvia Janetzki, Ilan Kirsch, Giuseppe V. Masucci, Paul B. Robbins, Senthamil R. Selvan, Howard Z. Streicher, Jenny Zhang, Lisa H. Butterfield, Magdalena Thurin

**Affiliations:** 1 Department of Epidemiology and Biostatistics, College of Public Health, The University of Georgia, 101 Buck Road, Athens, 30602 GA USA; 2 NanoString, Inc., 530 Fairview Avenue N, Seattle, WA 98109 USA; 3 Janssen Research & Development, LLC, Spring House, PA 19477 USA; 4 Nodality, Inc., 170 Harbor Way, South San Francisco, 94080 CA USA; 5 ZellNet Consulting, Inc., 555 North Avenue, Fort Lee, 07024 NJ USA; 6 Adaptive Biotechnologies, Inc, 1551 Eastlake Ave. E., Seattle, WA 98102 USA; 7 Department of Oncology-Pathology, Karolinska Institutet, 171 76 Stockholm, Sweden; 8 Pfizer, San Diego, CA USA; 9 Omni Array Biotechnology, 15601 Crabbs Branch Way, Rockville, 20855 MD USA; 10 National Cancer Institute, National Institutes of Health, 9609 Medical Center Drive, Bethesda, 20892 MD USA; 11 Covaris Inc., 14 Gill St, Woburn, MA 01801 USA; 12 Department of Medicine, Surgery and Immunology, University of Pittsburgh Cancer Institute, 5117 Centre Avenue, Pittsburgh, PA 15213 USA; 13 National Cancer Institute, Cancer Diagnosis Program, DCTD, National Institutes of Health, 9609 Medical Center Drive, Bethesda, 20892 MD USA

**Keywords:** Biomarker, Immunotherapy, Cancer, Assay, Validation, Regulatory

## Abstract

There is growing recognition that immunotherapy is likely to significantly improve health outcomes for cancer patients in the coming years. Currently, while a subset of patients experience substantial clinical benefit in response to different immunotherapeutic approaches, the majority of patients do not but are still exposed to the significant drug toxicities. Therefore, a growing need for the development and clinical use of predictive biomarkers exists in the field of cancer immunotherapy. Predictive cancer biomarkers can be used to identify the patients who are or who are not likely to derive benefit from specific therapeutic approaches. In order to be applicable in a clinical setting, predictive biomarkers must be carefully shepherded through a step-wise, highly regulated developmental process. Volume I of this two-volume document focused on the pre-analytical and analytical phases of the biomarker development process, by providing background, examples and “good practice” recommendations. In the current Volume II, the focus is on the clinical validation, validation of clinical utility and regulatory considerations for biomarker development. Together, this two volume series is meant to provide guidance on the entire biomarker development process, with a particular focus on the unique aspects of developing immune-based biomarkers. Specifically, knowledge about the challenges to clinical validation of predictive biomarkers, which has been gained from numerous successes and failures in other contexts, will be reviewed together with statistical methodological issues related to bias and overfitting. The different trial designs used for the clinical validation of biomarkers will also be discussed, as the selection of clinical metrics and endpoints becomes critical to establish the clinical utility of the biomarker during the clinical validation phase of the biomarker development. Finally, the regulatory aspects of submission of biomarker assays to the U.S. Food and Drug Administration as well as regulatory considerations in the European Union will be covered.

**Electronic supplementary material:**

The online version of this article (doi:10.1186/s40425-016-0179-0) contains supplementary material, which is available to authorized users.

## Background

Rapid advances in our understanding of the fundamental biology of cancer and the integral role of the immune response to tumor progression are changing drug development and clinical practice. Therapies that modulate the immune system are proving effective across a range of cancers, such as melanoma, non-small cell lung cancer (NSCLC), renal cell carcinoma and bladder cancer [1–4]. In parallel, emerging diagnostic technologies are making it possible to query multi-dimensional analytes, including multiplexed DNA, RNA, protein, and cellular infiltrate to characterize immune responses in the tumor. These advancements are providing exciting opportunities for the development of new treatment strategies that use cancer biomarkers to identify patients whose cancer may be more likely to respond to specific immunotherapies and subsequently targeting these therapies to pre-selected patients. Though, amidst the promise, there is also concern that without careful attention to clinical validation and regulatory requirements, these biological insights will not translate into effective treatments for patients.

The Society for Immunotherapy of Cancer (SITC) Immune Biomarker Task Force reports (Additional file 1) provide a wide range of discussions of technologies for biomarker development, specifically in the context of biomarkers with the potential to predict response to immunotherapies. Additionally, Volume I of this two-volume series is focused on pre-analytical and analytical validation of biomarkers in this context, providing examples of assays and recommended guidance for the early phases of biomarker development. The current volume, Volume II, discusses aspects of clinical validation process and regulatory consideration related to these late stages of biomarker development. While these different aspects of biomarker development are distinct and usually performed by different teams of researchers (because they require different areas of expertise), they are part of a continuum. Therefore, it is imperative to start thinking about clinical validation and regulatory requirements early in the biomarker development process. Overall, we believe that the content in both Volumes I and II is critical to understanding the entire process, from biological discovery to clinical application of a predictive biomarker.

**Fig. 1:**
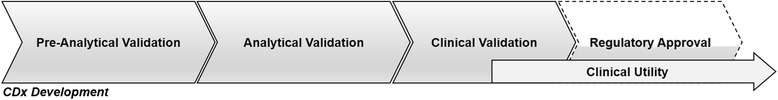
The biomarker development process can be schematically divided into sequential phases, including preanalytical and analytical validation, clinical validation, regulatory approval, and demonstration of clinical utility

After the analytical validity of a biomarker assay is established, as described in Volume I, the test must be evaluated to assess its clinical performance both in predicting the clinical outcome of interest, i.e., clinical validation — as well as in resulting in patient outcomes improvement, i.e., clinical utility (Fig. 1).

This volume describes clinical validity and utility requirements for predictive biomarkers, and discusses the variety of challenges encountered during the clinical validation process, particularly with complex multiplex or omics-based assays.

We discuss both retrospective and prospective validation of clinical utility of biomarkers, including different clinical design options for prospective validation trials. Recommended criteria for the clinical validation and validation of clinical utility steps for biomarkers development are provided. We also address the regulatory requirements for biomarkers by the U.S. Food and Drug Administration (FDA), including in vitro diagnostic tests and companion diagnostics (CDx). In addition, comparisons are made with the regulatory requirements of the European Union (EU) system.

## Clinical validation

### Clinical validity and utility

The final stage in the development of a biomarker predictive of clinical outcome is the assessment of its clinical validity and utility through the application of the analytically validated assay within a clinical trial, with multiple design options depending on the intended use of the test and availability of specimens from previous clinical trials.

Clinical validity relates to the observation that the predictive assay reliably divides the patient population(s) of interest into distinct groups with divergent expected outcomes to a specific treatment [5, 6]. The criteria for validation are defined by the nature of the question that the biomarker is intended to address (i.e., fit-for-purpose). A predictive biomarker needs to demonstrate the association with a specific clinical endpoint (e.g., survival or tumor response) in pre-treatment samples from patients that have been treated or exposed to a uniform treatment intervention. For example, the programmed cell death-1 protein ligand (PD-L1) immunohistochemistry (IHC 22C3 pharmDx) test was approved as a CDx to pembrolizumab as a single agent in second-line NSCLC. The test was used to determine patient eligibility in a single arm study KEYNOTE 001 [2].

In this study, the proportion of tumor cells expression of PD-L1 ≥50 % was shown to be associated with the clinical endpoint of durable response (and accelerated approval was obtained from the FDA for that indication). In contrast, the PD-L1 IHC 28–8 pharmDx test was approved by the FDA as a complementary test to another PD-1 inhibitor nivolumab (Bristol-Myers Squibb) in the non-squamous NSCLC and melanoma patient populations. The test was not used for patient selection in the randomized phase III trial, which compared single agent nivolumab to docetaxel (standard of care); it was developed in a retrospective fashion to inform on the risk vs. benefit for patient subsets defined by tumor PD-L1 positivity. The third and most recently approved assay is also a complementary diagnostic that was approved for patients with metastatic urothelial cancer considering treatment with the anti-PD-L1 therapy atezolizumab. An association between PD-L1 expression in the tumor microenvironment and patient overall survival was observed in the nivolumab arm but not in the docetaxel arm, which illustrated the predictive value of the assay [7].

**Fig. 2:**
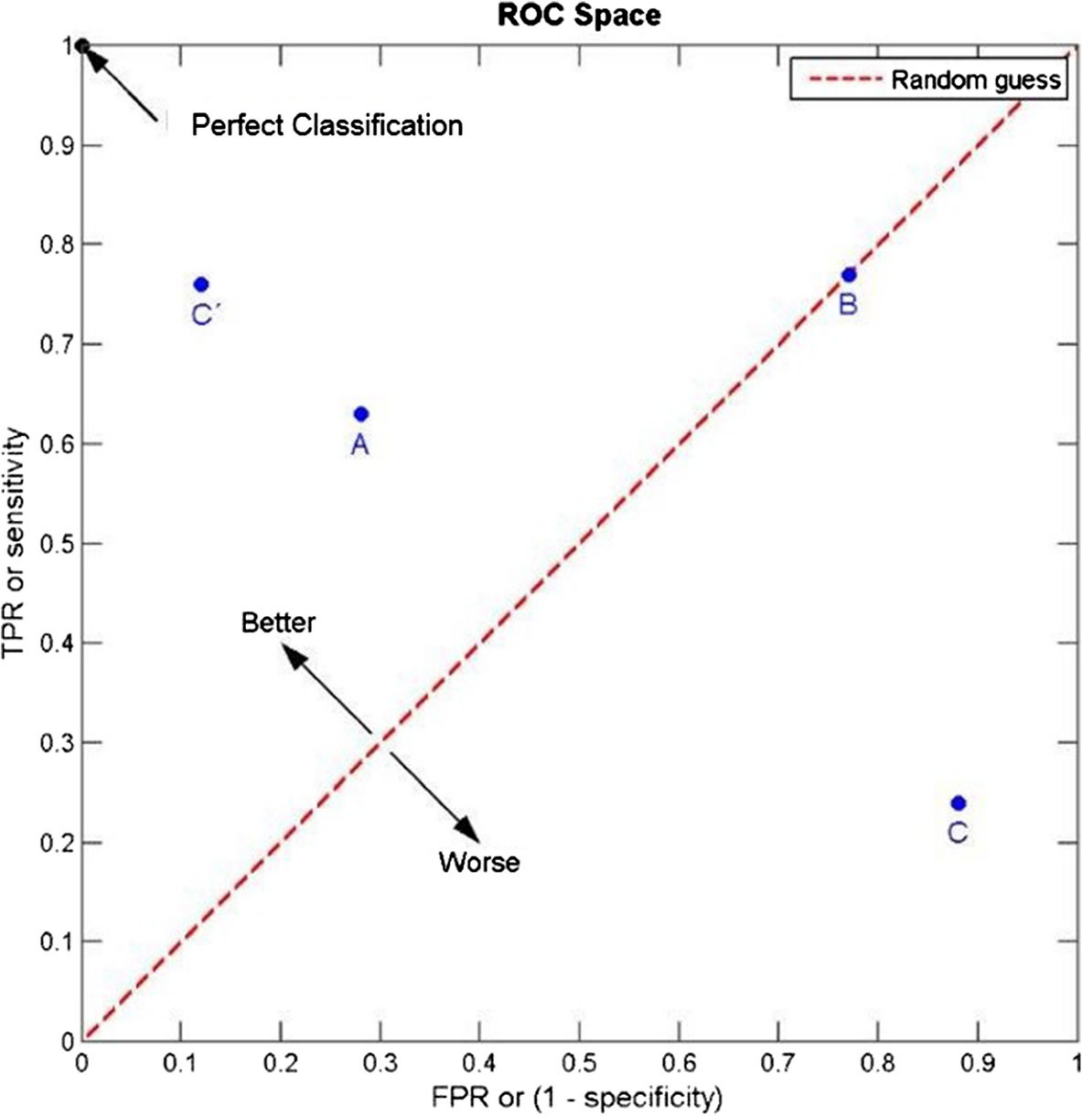
The clinical sensitivity and specificity of a biomarker assay must be demonstrated through robust receiver operative characteristics (ROC) curves. As illustrated, an ROC curve is a plot that captures true positive rate (TRP) against false positive rate (FRP) at various threshold settings

Among the parameters required for clinical validation of a test are the data regarding the clinical sensitivity/specificity, reproducibility, analyte stability, and cutoff of the assay (Table 1) [8, 9]. The clinical sensitivity and specificity of the assay must be demonstrated through robust receiver operating characteristics (ROC) curves that provide support for the cut points, established using appropriate statistical analysis to identify responders vs. non-responders. The ROC curve is essentially a plot that captures true positive rate (TPR) against false positive rate (FPR) of an assay (Fig. 2). The optimal cut point is the point on the curve corresponding to a FPR and TPR best suited to the clinical context. As an example, the KEYNOTE 001 study (*n* = 496), demonstrated a positive correlation between PD-L1 expression and treatment outcome in patients with advanced NSCLC treated with pembrolizumab. In this study, approximately one-third of the patients were assigned to a training group and two-thirds of the patients were assigned to a validation group. The data from the training group were used to define the clinical cutoff for the PD-L1 IHC. Based on the ROC analysis, positive predictive value (PPV), negative predictive value (NPV) and PD-L1 prevalence in the training set, a proportion score of ≥50 % was selected for validation in the testing set [2]. In contrast, in the CheckMate 057 study, which evaluated the benefit of nivolumab versus docetaxel in an unselected population of advanced NSCLC, a retrospective analysis of tumor specimens using the anti-PD-L1 28–8 pharmDx assay showed a correlation between the level of PD-L1 expression and all efficacy endpoints at an expression level of >1 % [10].

**Table 1:** Parameters for evaluating clinical validity of a predictive biomarker

Parameter	Definition
Clinical sensitivity	Sensitivity of the biomarker, is the ability of a biomarker or a change in biomarker to predict a meaningful change in a clinical endpoint. Sensitivity describes the relationship between the magnitude of change in the biomarker and the magnitude of change in the clinical endpoint. For example, a 50-unit increase in OncotypeDX recurrence score (RS-PCT/50) was associated with an estimated increase of 2.87 in hazard ratio (Tang et al., 2011 [21]) of distant recurrence (DRFI endpoint) in tamoxifen-treated patients.
Clinical specificity	Specificity of the biomarker, referred to as the ability of a biomarker or a change in biomarker to distinguish patients who are responders to an intervention from those who are non-responders in terms of changes in clinical endpoints. For example, the estimated hazard ratio for chemotherapy (no chemotherapy divided by chemotherapy) in the low OncotypeDX recurrence score (RS) group was 1.31 versus 0.26 in the high RS group (Tang et al., 2011 [21]), where the outcome is DRFI.
Probability of false positives	False positives occur when a desired change in a biomarker is not reflected by a positive change in a clinical endpoint or even worse, is associated with a negative change in a clinical endpoint. An example of a false positive is the detection of elevated levels of the functional or biochemical marker in the absence of clinical response to treatment. For example, a tumor that has expressed PD-L1 on the tumor cells, but does not respond to targeted anti-PD-L1 immunotherapy, is a false positive.
Probability of false negatives	False negatives occur when no change or a small observed change in a biomarker fails to signal a positive, meaningful change in a clinical endpoint; for instance a tumor that does not express PD-L1 but does respond to anti-PD-L1 immunotherapy is a false negative.
AUC	Area under ROC curve. AUC is used to compare different tests, i.e., an AUC value close to 1 indicates good discrimination, whereas an AUC of 0.5 provides no useful information regarding the likelihood of response.
ROC analysis	A graphical approach for showing accuracy across the entire range of biomarker concentrations. ROC, use to set cut points, is essentially a plot that captures true positive rate against false positive rate of an assay.
Cut point	The sensitivity and specificity of the assay must be demonstrated through robust ROC curves that provide support for the cut points established to identify responders vs. non-responders.
Hazard ratio	Chance of an event (e.g., disease recurrence, death) occurring in the treatment arm divided by the chance of the event occurring in the control arm, or vice versa.
Relative risk	Ratio of the probability of an event (e.g., disease recurrence, death) occurring in treated group to the probability of the event occurring in the control group.

Statistically, several approaches can be applied for clinical validation of an assay. Internal validation can be achieved by using a study population that reflects the target population in which the test will be used. The study population is divided into two independent groups of specimens. One of these groups is the “training set,” i.e., the set of samples used to identify and characterize the “biomarker” (if single analyte) or to build a mathematical model or algorithm (in case of multi-variate assays). The second sample group is “the validation set” that is used to test whether the external validity of the biomarker/model is maintained in a sample cohort—independent from the training set. Cross-validation using multiple mutually exclusive “training” and “validation” samples can be carried out in order to compensate for overfitting. But, neither internal validation nor cross-validation is adequate for clinical validation. External validation on an independent dataset, or multiple independent datasets, is required. Ultimately, the clinical performance of any predictive biomarker requires external validation for regulatory approval. In all approaches, the number of patients in the group for validation must be large enough to provide sufficient statistical power at a 5 % significance level [11].

Biomarker validation must also be sufficiently robust to achieve a high level of performance in routine clinical samples. Ultimately, clinical decisions must be based on the assays and cut points derived from samples that reflect the target population.

#### Challenges in clinical validation

##### Biomarker characteristics: single analyte versus multivariate assays

Predictive markers can be defined as a single biomarker or signature of markers that separate different populations with respect to the outcome of interest in response to a particular treatment. A distinguishing characteristic of multivariate assays is that computational methods are applied to the high-dimensional data, e.g., gene expression profiling using NanoString, single cell network profiling (SCNP), or fluorescence-activated cell sorting (FACS), to build mathematical models, often from a subset of the measured variables that have been identified through data-driven selection. This is in contrast to the single analyte molecular tests based on pre-specified, biologically driven variables, such as mutations in genes (BRAF) or protein expression targeted by a specific therapeutic agent (HER2/neu expression). Single analyte tests must be based on well-established analytical performance. Similarly, multianalyte assays based on complex computational models must also achieve robust analytical performance but pose additional challenges that are distinct from the single analyte realm.

In the development of multianalyte predictive or prognostic assays, the discovery phase includes complete definition of the computational algorithm to be optimized and validated in independent sample cohorts. At this point, the fully specified computational algorithm should be locked down, recorded and no longer changed before it is applied in the validation of clinical utility step. Statistical and bioinformatics evaluation needs to occur throughout both development stages (discovery and validation). What defines adequate validation is much different in the early phases of biomarker development compared with the later phases of development. Early on, the focus is on basic biological and bioinformatics data processing, technical reproducibility, and technical sources of variation. However, for successful development of clinically useful test, it is critical that this focus shifts toward the evaluation of the patient-to-patient variation in the levels of the underlying biological analytes. The final clinical utility of the biomarker is often limited by natural biological variation that is present in complex systems rather than technical assay challenges, which may be overcome by novel developments in assaying specimens.

##### Bias

One of the most common problems in clinical validation is bias or systematic error that is the source of results unrelated to clinical outcomes and that are not reproducible. Sources of bias can include: i) differences in relevant demographic characteristics between training and testing sets, ii) differences in pre-analytic variables (sample handling, storage time, and variability arising from different collection protocols), and iii) divergence from assay protocols. These are critical issues often overlooked in the biomarker discovery process that are likely to be the single greatest reason why most biomarker discoveries fail to be clinically validated.

##### Overfitting

Computational methods are applied to generate functional algorithms for assays which measure multiple variables to predict clinical parameters such as patient outcome in response to treatment (e.g., NanoString and SCNP). These algorithms are vulnerable to overfitting. Overfitting can occur when large numbers of potential predictors are used to discriminate among a small number of outcome events. It can result in apparent discrimination (for example, between patients whose tumor responded or didn’t respond to a certain treatment) that is actually caused by chance and is, therefore, not reproducible. Thus, the importance of rigorously assessing the biological relevance and clinical reproducibility of the predictive accuracy of an assay is higher in the development of the computational model than for a single biomarker-based test.

To avoid being deceived by the overfitting phenomena, the algorithm that is derived in the group of samples defined as the ‘training set’ should be applied to an independent group of samples called the “validation set”, consisting of samples collected from patients who are not included in the training set. Typically, internal validation (also called cross-validation) is used to gauge how stringent one should be in selecting potential predictors to include in the model and to reduce this number to a small, robust core signature. Correct cross-validation requires strict adherence to the principle of no “information leak” between the training set and the validation set, so that at each cross-validation step the predictor is constructed “from scratch.” When performed correctly, statistical cross-validation is a powerful tool for estimating biomarker performance [12]. As the assay moves towards clinical implementation, the need for external validation on independent datasets becomes critical to assess the impact of technical sources of variation and bias that may not be present when a single study dataset is considered in isolation.

##### Appropriateness of the statistical methods used to build the predictor model and to assess its performance

The high dimensionality of -omics data and the complexity of many algorithms used to develop omics-based predictors including immunomics, present many potential pitfalls if proper statistical modeling and evaluation approaches are not used. Various statistical methods and machine learning algorithms are available to develop models, and each has its strengths and weaknesses. With the development of next generation sequencing (NGS) and other molecular technologies, the dimensionality and complexity of potential diagnostics has greatly increased; in particular, storing the resulting terabytes of biological data becomes challenging.

**Fig. 3:**
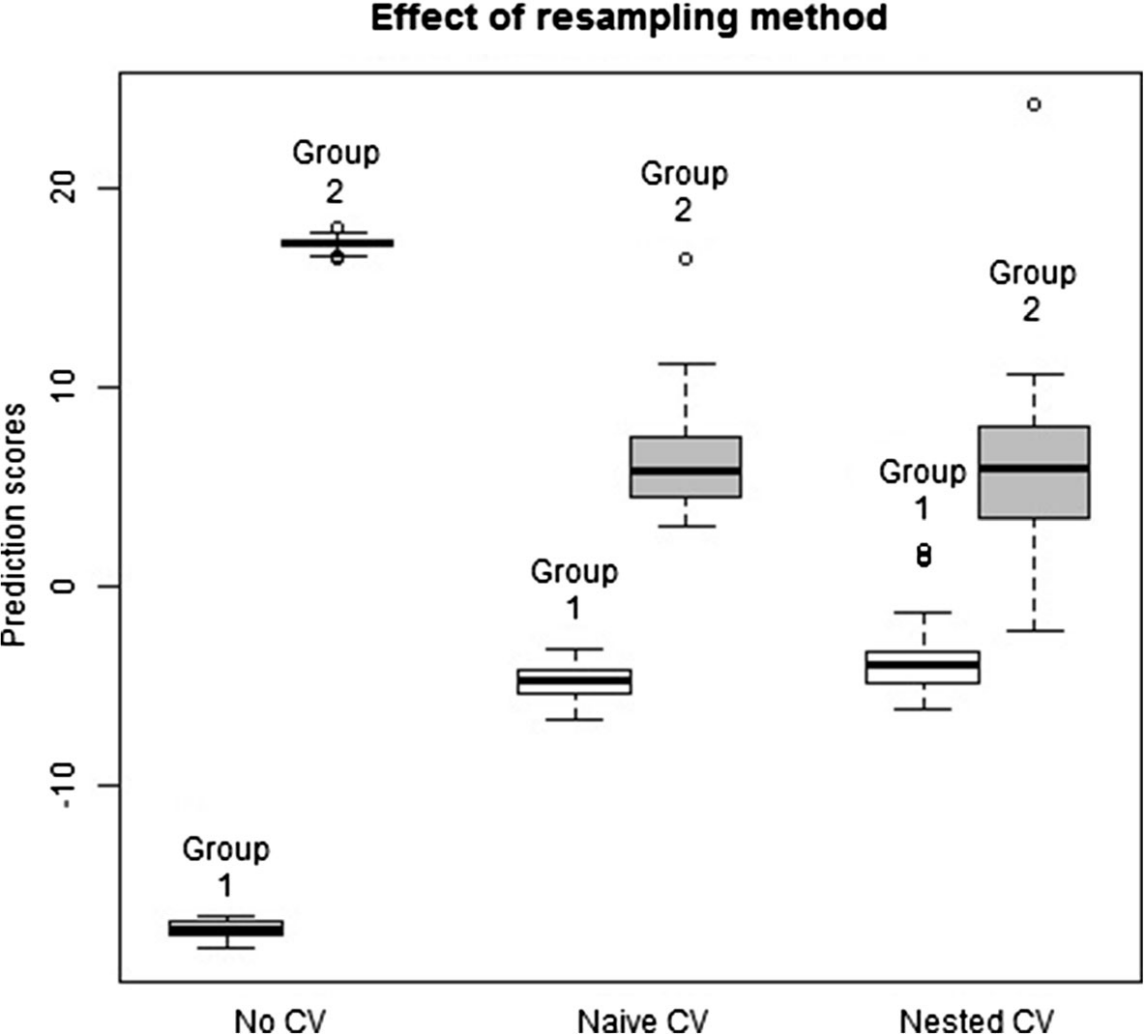
The impact of improper resampling shown on an RNASeq dataset [13, 14]. Samples are classified into Group 1 (CEU, *n* = 69 samples) versus group 2 (YRI, *n* = 60 samples) using the lasso logistic regression classifier as implemented in the glmnet package [36]. The “No CV” case did not use cross-validation to pick a value for the tuning parameter, instead using a fixed value 4e-9. The “naïve CV” method used naïve, non-nested cross-validation to pick the tuning parameter. The “nested CV” method used nested cross-validation to pick the tuning parameter, so that there was never any overlap between the data used to develop the predictor and the data used to estimate and evaluate the prediction scores. The accuracy estimated from the correct nested CV method is 95 %, and from each of the other methods is 100 %, the difference representing bias due to erroneous resampling

As a relevant sample dataset to illustrate the impact of improper resampling**,** RNA-Seq data were used to evaluate the transcriptomes of 60 HapMap individuals of European descent [13] and 69 unrelated HapMap Nigerian individuals [14]. Raw data were processed as described previously [15]. Subsequently, lasso logistic regression [16] was specified as the classifier development algorithm. The lasso uses a tuning parameter to select features for the model. The statistically correct analysis uses nested cross-validation to estimate the prediction scores and accuracy. This is compared to no cross-validation and naïve (non-nested) cross-validation in Fig. 3. As can be seen from the figure, both no cross-validation and naïve cross-validation result in apparent perfect separation of the two groups’ prediction scores, and 100 % accuracy. However, the unbiased nested cross-validation results in overlap between the groups and a more realistic and unbiased estimated classification accuracy of 95 %.

### Recommendations — criteria  for the clinical validation of a robust predictive marker

- For multi-analyte classifiers, internal validation should be performed for the model development, tuning, and validation.
- External validation is critical. In external validation, a fully “nailed down” predictor is applied to a novel dataset from a source that is different (typically a different laboratory and clinic) and most critically a non-overlapping set of patients.
- Many modern statistical methods involve extensive resampling of a training set during the model development and complex averaging over a large and varied set of prediction models. These methods include statistical boosting and bagging as well as Bayesian model averaging. The resulting “black box” nature of these algorithms makes them problematic to evaluate. As they move towards the clinic, these should be simplified into more transparent models, such as linear or generalized linear models.
- Cut points used for classification and stringency levels used for model tuning need to be specified prior to external validation on independent datasets.

### Validation of clinical utility

The clinical utility is a measure of whether clinical use of the test improves patient outcomes for a specific indication, i.e., the final results of a test must support specific decisions/actions that result in improvement of patient overall survival in order to have clinical utility. The clinical utility step for predictive marker validation is carried out under the assumption that the methods used for assessment of the biomarker are established and the clinical validation results confirm the predictive ability of the marker(s). To assess the clinical utility of the predictive assay, adequate and well controlled prospective clinical trials or retrospective analysis of collected specimens from completed trials with appropriate justification may be used. These studies must i) define standardized relationships between therapeutic intervention and response and ii) provide estimates of the magnitude of benefit. Examples of such studies in immune-oncology are the trials that supported the regulatory approval of the two different IHC assays detecting PD-L1 expression in NSCLC tissue linked to the use of pembrolizumab and nivolumab [2, 10].

#### Clinical trial design for assay clinical validation and validation of clinical utility

Design of a clinical trial for definitive evaluation of any predictive test must begin with a clear statement of the target population and the intended clinical use. In the case of banked clinical trial specimens used in a retrospective study, the protocol should be amended, or a formal proposal submitted to the gatekeepers of the bank, prior to sample analysis. Information about the anticipated distribution of test results in the population and the magnitude of the expected effect or benefit from use of the test should be gathered from preclinical or retrospective hypothesis generating studies. On the basis of that information, it should be determined whether it will be feasible to design a trial or clinical study of sufficient size to demonstrate clinical utility [17].

**Fig. 4:**
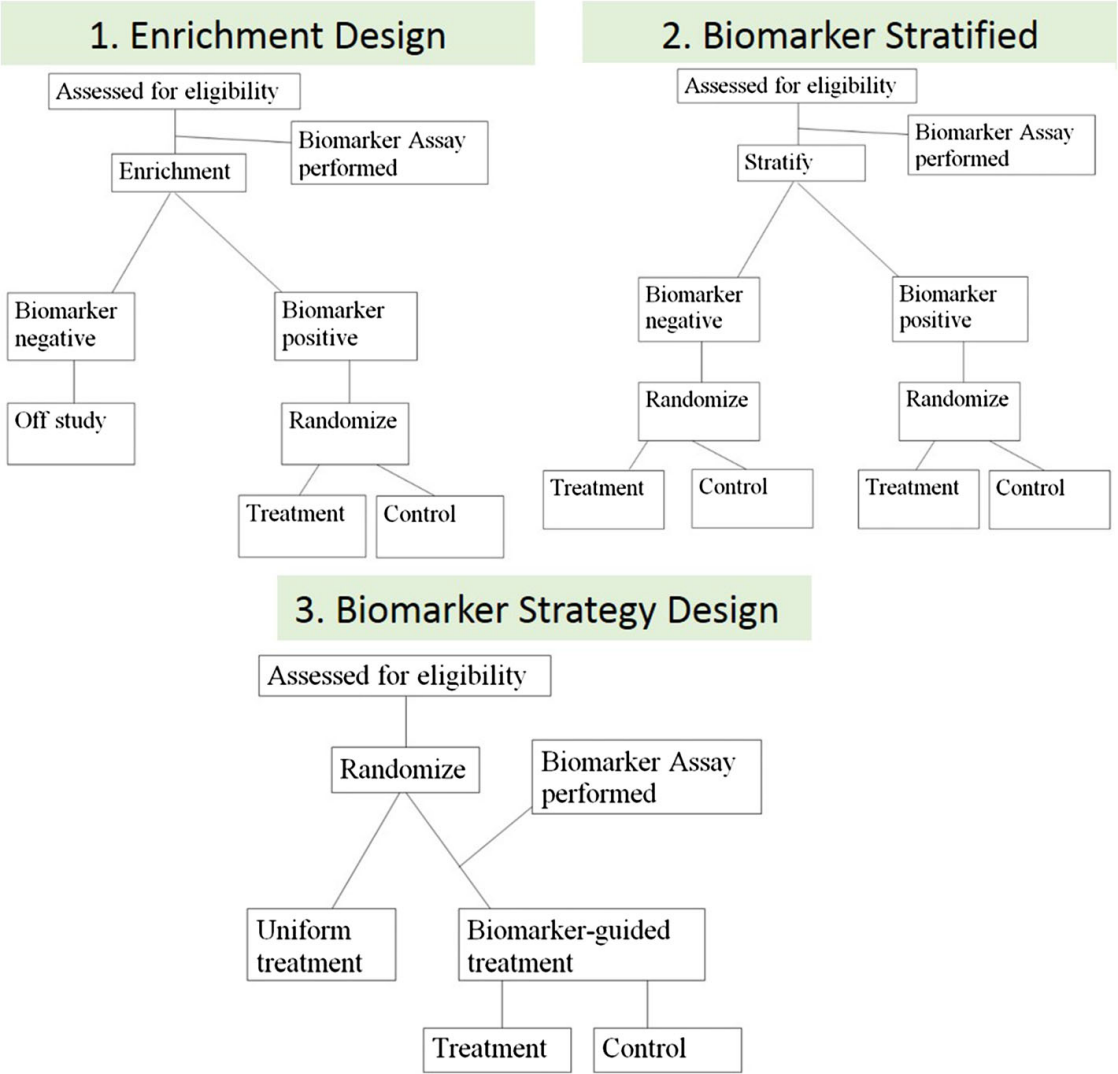
There are three basic phase III design options for assessing the ability of a biomarker. The enrichment design includes only patients who are positive for the biomarker in a study evaluating the effect of a new therapy (1). In the biomarker stratified design, all patients, independent of biomarker results, are enrolled and randomized to treatment and control groups within each of the biomarker positive and negative groups to ensure balance (2). Finally, in the strategy design, patients are randomized between no use of the biomarker (all patients receive standard therapy on that arm) and a biomarker-based strategy where biomarker-negative patients receive standard therapy and biomarker-positive patients receive the new therapy (3)

There are three basic phase III design options that are frequently considered for assessing the ability of a biomarker to identify a subgroup of patients who will benefit from or will not benefit from a new therapy, and therefore should be avoided (Fig. 4). These are classified broadly into three categories: 1) the enrichment design, 2) the stratified design, and 3) the strategy design.

In the enrichment design, only patients who are “positive” for the biomarker (above a specific cutoff) are included in a study evaluating the effect of a new therapy (Fig. 4.1 ). This is the design used in the trial which led to the approval of PD-L1 22C3 pharmDx as a CDx for pembrolizumab in advanced NSCLC [2]. Another example is an enrichment design strategy for enrolling only human epidermal growth factor receptor 2 (HER2)–positive patients. This study demonstrated that trastuzumab combined with paclitaxel after doxorubicin and cyclophosphamide significantly improved disease-free survival (DFS) among women with surgically removed HER2/neu-positive breast cancer [18]. This design results in an enrichment of the study population, with a goal of understanding the safety, tolerability, and clinical benefit of a treatment in the subgroup(s) of the patient population defined by a specific marker status. If marker status is based on an underlying continuous measurement, then multiple unique cutoffs may be evaluated using an appropriate multiple comparison procedure. This approach can answer the question of whether biomarker-positive patients benefit from the new therapy, but it cannot be used to empirically assess whether biomarker-negative patients might benefit as well. Therefore, preliminary evidence to suggest that patients without the marker do not benefit from new therapy needs to be established for enrichment trial to be appropriate. Also, it does not allow for distinction between predictive and prognostic biomarkers.

The stratified study design enrolls all patients, independent of biomarker status, but then patients are randomized to treatment groups separately within each of the biomarker positive and negative groups to ensure balance of the treatment arms within each group (Fig. 4.2 ). In this study design, the biomarker guides the analysis but not the treatment. This approach provides maximum information about the ability of the biomarker to identify patients who will benefit/not benefit from the new therapy, i.e., allows distinction between predictive and prognostic biomarkers. This maximum information is gained at some cost, since this design also typically requires larger sample sizes. However, a stratified design does not allow the biomarker to influence what treatment a patient receives in the trial; this can be an advantage in a situation where there is some uncertainty about the strength of a biomarker’s performance, but this can also be considered unethical if strong biologic rationale exists that suggests a lack of efficacy in the biomarker negative patient population. Therefore, when a trial randomizes “test-negative” patients (i.e., below pre-defined assay cutoff), there should be provisions for aggressive futility monitoring so that the trial can be stopped early if substantial evidence emerges that these patients are not benefitting from the new therapy. An example of the marker-by-treatment-interaction design is the phase III biomarker validation study, known as MARVEL (Marker Validation of Erlotinib in Lung Cancer), of second-line therapy in patients with advanced NSCLC randomly assigned to pemetrexed or erlotinib based on epidermal growth factor receptor (EGFR) status as measured by fluorescence *in situ* hybridization (FISH) [19].

The strategy design randomizes patients between no use of the biomarker (all patients receive standard therapy on that arm) and a biomarker-based strategy where biomarker-negative patients are directed to standard therapy and biomarker-positive patients are directed to the new therapy (Fig. 4.3 ). A strategy design in the context of a single biomarker is particularly inefficient because patients who are negative for the biomarker will receive standard therapy regardless of whether they are randomized to use the biomarker. This results in a reduction in the effective sample size and loss of power. Due to this inefficiency, this strategy design is generally not recommended in a simple single-biomarker setting [20]. An example of the strategy design is the trial to test whether excision repair cross-complementing 1 (ERCC1) gene expression is a predictive biomarker associated with cisplatin resistance in NSCLC. In the ERCC1 trial, patients were randomly assigned to the control arm that received cisplatin + docetaxel or the biomarker-strategy arm that switched patients classified as cisplatin resistant to gemcitabine + docetaxel regimen while treating those nonresistant with standard cisplatin + docetaxel [21].

A clinical trial to evaluate the clinical utility of an omics test should be conducted with the same rigor as a clinical trial to evaluate a new therapy. This includes development of a formal protocol clearly detailing pre-specified hypotheses, study methods, and a statistical analysis plan. In some instances, a candidate predictive test for an existing therapy can be evaluated efficiently by using a prospective-retrospective design, in which the test is applied to archived specimens from a completed trial and the results are compared with outcome data that have already been or are currently being collected. The “retrospective” aspect of this design requires that the assay can in fact be performed reliably on stored specimens. The ‘prospective’ aspect of the design refers to the care taken prior to sample analysis to ensure the following:

- The patients in the trial are representative of the target patient population expected to benefit from the test.
- There is a pre-specified statistical analysis plan.
- Sufficient specimens are available from cases that are representative of the trial cohort and intended use population to fulfill the sample size requirements of the pre-specified statistical plan, and those specimens have been collected and processed under conditions consistent with the intended-use setting. For example, NSABP B-14 and B-20 samples were used in order to validate the 21-Gene Recurrence Score Assay (Oncotype DX) in breast cancer [22]. Another example of a marker that has been successfully validated using data collected from previous randomized controlled trials is KRAS as a predictor of efficacy of panitumumab and cetuximab in advanced colorectal cancer [23].

In general, two such prospective-retrospective studies producing similar results will be required to have confidence that the clinical utility of the test has been established. While retrospective validation may be acceptable as a marker validation strategy in select circumstances, the gold standard for predictive marker validation continues to be a prospective randomized controlled trial as discussed above.

The measurement of clinical utility of cancer immunotherapies when compared to other anti-cancer approaches might require different criteria. Specifically, the RECIST and WHO criteria, which were not developed specifically for immunotherapy but for cytotoxic therapies, may not capture antitumor responses induced by immunotherapeutic approaches adequately. Specifically, delayed tumor responses improving over months are common in patients responding to immunotherapy approaches. In response to these observations, new immune response criteria have been developed [24]. The delayed separation of Kaplan-Meier curves in randomized immunotherapy trials can have effects on the development and validation of predictive biomarkers of immunotherapy clinical benefit. This may particularly be a problem for log-rank test statistical approaches that weight all evaluation times equally; however, alternatives such as the Wilcoxon or Peto-Prentice weighting will tend to weight later times more and may ameliorate this effect. Also, in the context of Cox proportional hazards modeling, a time-varying coefficient model may be an effective methodology for modeling the effect of the therapy as it changes over time.

In conclusion, immunotherapies have emerged as the most promising class of drugs to treat patients with cancer with diverse tumor types; however, many patients do not respond to these therapies. Therefore, determining which patients are likely to derive clinical benefit from immune checkpoint agents remains an important clinical question and efforts to identify predictive markers of response are ongoing. The development and clinical validation of such predictive biomarkers require appropriate clinical studies in which the evaluation of the clinical utility of the biomarker is a pre-specified endpoint of the study. A variety of study designs have been proposed for this purpose. Although, the randomized biomarker stratified design provides the most rigorous assessment of biomarker clinical utility, other study designs might be acceptable depending on the clinical context. In this review, we have attempted to provide examples of the designs for predictive biomarker validation along with recommendations for important requirements for the clinical validation process that could aid development of clinically applicable biomarkers to predict response to immunotherapy.

### Recommendations — criteria for evaluating the performance of a predictive biomarker

- A study designed to assess the clinical validity of a predictive biomarker, must predefine (i.e., prior to sample analysis) the clinically meaningful performance metric(s) for the predictor (see below). In addition, the clinical setting (for example, disease type and stage, specimen format) must be similar to the intended-use setting of the predictive test.
- Guidelines have been developed for informative reporting of studies on the prediction of genetic risk and on prognostic as well as diagnostic markers and are applicable to a wide variety of predictive biomarkers, including biomarkers for cancer immunotherapy. Thus, these guidelines should be used during the planning and implementation of studies to evaluate predictive biomarkers.
- The choice of specific performance metric (for example, sensitivity and specificity, positive and negative predictive value, C-index, area under the ROC curve) and the benchmark performance level that must be attained is dependent on the intended clinical use. In order to sort out the predictive versus prognostic value of a biomarker from a stratified design, it is necessary to evaluate the effect of an interaction between the marker and the treatment. Only specific interactions will result in a marker that can improve patient outcomes in the target population. Key ideas in this developing area of statistical research are reviewed in Janes et al. (2013) and can be used as a reference [25].
- Demonstration that a predictor’s output is statistically associated with the clinical endpoint is not sufficient evidence of acceptable performance. Although the presence of such an association may establish the clinical validity of the test, statistical significance (for example, P <0.05) does not always translate into a clinically meaningful association or provide clinically useful, or actionable, information. To establish clinical utility, as opposed to clinical validity, there must be evidence suggesting that the use of the test is likely to lead to a clinically meaningful benefit to the patient beyond current standards of care**.**

## Regulatory considerations for assays submission to FDA

With increasing understanding of the molecular basis of cancer, research and clinical laboratories are developing and implementing a variety of molecular diagnostic tests to guide cancer therapy including immunotherapy. Before introducing any new test into the market, the analytic and clinical performance characteristics of the assay must be validated. If the assay is developed as an in vitro diagnostic (IVD), then it must be approved/cleared by the FDA; if the assay is developed as a laboratory-developed test (LDT), only analytic validation is needed for commercialization. Understanding the regulatory approval process for IVDs to be used in making healthcare decisions is important for the development and performance assessment of any clinical diagnostic.

### Regulation of diagnostic tests in the United States

For this article, we will focus only on IVD tests that are regulated by the FDA’s Center for Devices and Radiological Health (CDRH). IVDs are defined as medical devices in section 210(h) of the Federal Food, Drug, and Cosmetic act. The classification of an IVD (or any medical device) into one of the three classes — class I, class II, or class III — is largely based on the level of risk: low-, moderate-, and high-risk, respectively. Risk determination for an IVD is primarily based on the harm to a patient that might be incurred as a result of an incorrect test measurement when the test is used as intended, although it can include other types of risks (Table 2). For example, a false-negative test result may alter medical management and a false-positive test result may result in an invasive medical procedure.

**Table 2:** FDA risk classification for medical devices

FDA Classification	Definition
Class I	Minimal potential for harm to patients and is subject to the least amount of regulatory controls.
Class II	Higher risk to patients and requires greater regulatory controls to provide assurance of safety and efficacy.
Class III	Highest risk devices that typically sustain or support life, are implanted, or present potential unreasonable risk of illness or injury. This class has the highest level of regulatory control and therefore must be approved by the FDA before being marketed.

- The lowest risk tests (class I) are those for which general controls (e.g., registration, listing and implementation of a quality system for the product) are sufficient to provide reasonable assurance of the safety and effectiveness of the device and typically do not require a premarket submission to the FDA [26].
- Moderate risk (class II) tests are reviewed by the FDA through the premarket notification process otherwise known as the 510(k) pathway, relying on “special controls” to provide assurance of safety and effectiveness. This pathway involves submitting a 510(k) premarket notification demonstrating that the test is substantially equivalent to a legally marketed (predicate) device already on the market.
- Class III devices require more rigorous premarket review by the FDA through the submission of a premarket approval application (PMA), where the sponsor must demonstrate, through analytical and clinical performance studies that the device is safe and effective for use in the intended population. The PMA process is generally used for novel and high-risk devices and requires FDA approval prior to marketing.

The FDA considers tests predictive of response to specific drugs including CDx that identify patients who are most likely to benefit from a particular therapeutic product as the highest risk class of IVDs (class III). These tests present significant risk due to the likelihood of harm to the patient if the diagnostic result is incorrect and therefore must be reviewed by the FDA. Predictive biomarker tests that are used to select patients for enrollment into a clinical study must be carried out either in Clinical Laboratory Improvement Amendments (CLIA) laboratories that are certified by the state in which they reside or by a Centers for Medicare and Medicaid Services (CMS)-approved accrediting institution such as the College of American Pathologists (CAP). Hospital laboratories and some university core laboratories may have such certifications in addition to some commercial laboratories.

A variety of tests including predictive tests have been developed and used in CLIA certified labs as LDTs (or “homebrew tests”) without FDA review, due to the agency’s longstanding policy of enforcement discretion for LDTs. However, in October 2014, the FDA announced that it intends to enforce device regulations for LDTs [27], with the goals of assuring safety and effectiveness. This requires adverse event reporting, removal of unsafe devices from the market, and assessing quality manufacturing of devices. The FDA will focus initially on high complexity assays that use multiple markers and mathematical algorithms to determine clinical validity of the test result.

### Companion diagnostics (CDx)

**Fig. 5:**
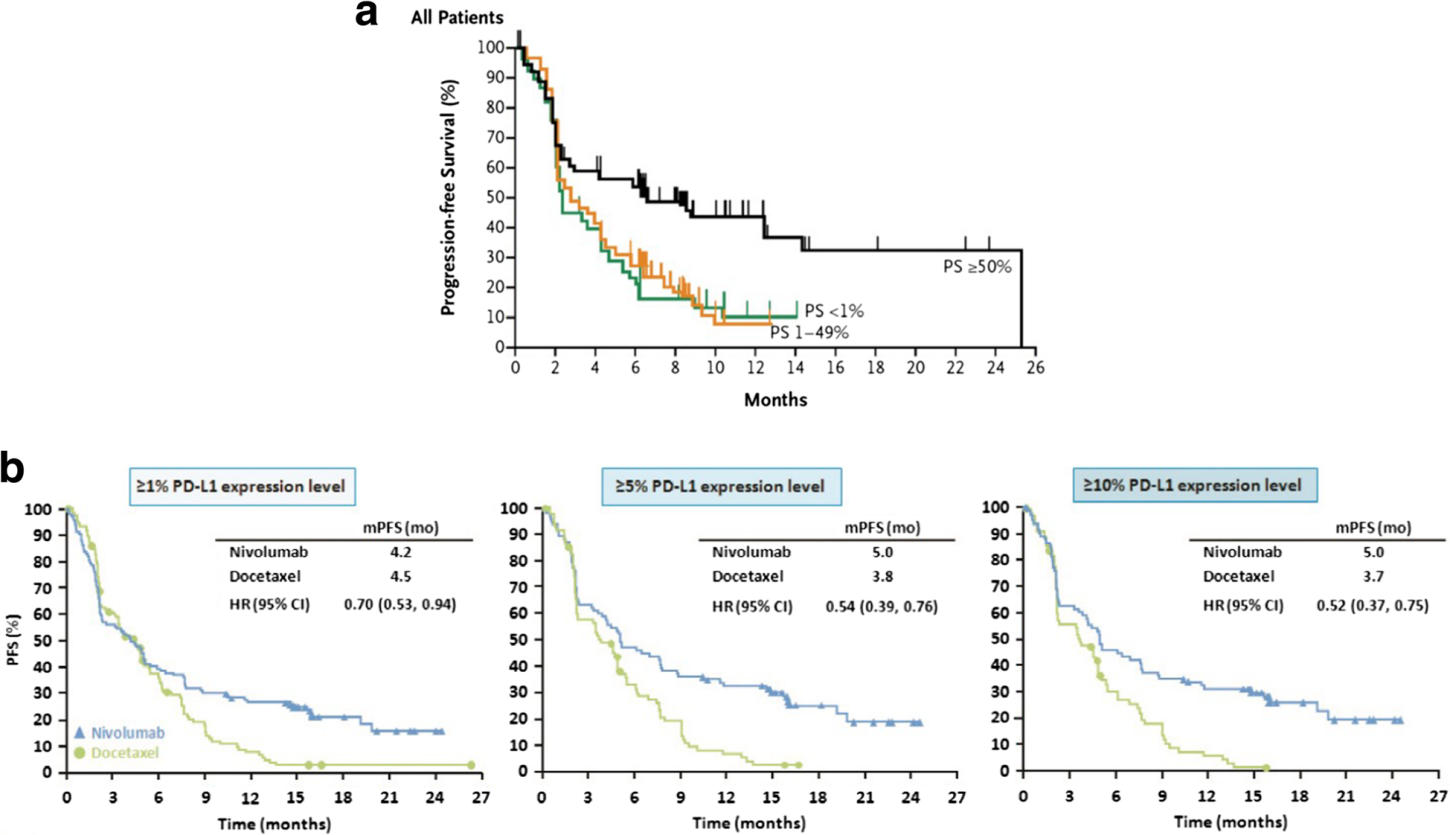
Representative survival curves illustrating the different clinical scenarios involved in the FDA approval of pembrolizumab using the PD-L1 22C3 PharmDx assay (**a**) vs. nivolumab using the PD-L1 28–8 PharmDx assay (**b**). For pembrolizumab administered in second-line NSCLC, panel **a** shows Kaplan–Meier estimates of progression-free survival according to the proportion score of the percentage of neoplastic cells with membranous PD-L1 staining. In this context, the biomarker was used as inclusion criteria to select the patient population in which the clinical activity of pembrolizumab was assessed. For nivolumab, PD-L1 expression was assessed retrospectively in prospectively collected tissue samples. Panel **b** illustrates Kaplan-Meier estimates of progress-free survival in patients receiving nivolumab or docetaxel by PD-L1 expression level. In this study, the test was not used for patient selection but to evaluate the interaction between PD-L1 expression and clinical benefit. Panel **a** from The New England Journal of Medicine, 2015, 372, 2018-2028 Edward B. Garon et al., Pembrolizumab for the Treatment of Non–Small-Cell Lung Cancer. Copyright © 2015 Massachusetts Medical Society. Panel **b** from The New England Journal of Medicine, 2015, 373, 1627-1639 Hossein Borghaei et al., Nivolumab versus Docetaxel in Advanced Nonsquamous Non–Small-Cell Lung Cancer, 373, 1627-1639. Copyright © 2015 Massachusetts Medical Society. Reprinted with permission from Massachusetts Medical Society

When a biomarker test is designed to be used in conjunction with specific treatment, the test is known as a CDx. Safety and efficacy of the new drug and of the CDx are typically demonstrated in the same clinical trial for both the drug and the test. Thus, for evaluating CDx, the FDA recommends that the development of the assay in parallel to its companion drug [28]. To date, this approach has been used to gain FDA approval for over 20 CDx in oncology. In particular, approval has been granted for tests for predicting response to targeted therapy drugs including tests for mutations (BRAF, C-KIT, and EGFR), protein expression (HER2/neu) or amplification (ALK), and more recently for three anti-PD-L1 IHC assays [29]. Approved drugs and their CDx refer to each other in their labels, as indicated in FDA guidance [30]. Currently, CDx are defined by FDA as devices that are necessary for the safe and effective use of a corresponding therapeutic product within its approved labeling, e.g. PD-L1 22C3 PharmDx (Dako) for pembrolizumab administration in second-line NSCLC. In this context, the biomarker was used as inclusion criteria to select the patient population in which the clinical activity of pembrolizumab was assessed. This resulted in the identification of a patient population highly enriched for pembrolizumab responders that formed the basis for the accelerated approval of the drug in this setting (Fig. 5a) [2].

### Complementary diagnostics

Complementary diagnostics are tests that, although not needed for the prescription of the corresponding therapeutic product, provide useful information on the drug risk/benefit in specific patient subsets, e.g., PD-L1 28–8 PharmDx for nivolumab in both non-squamous NSCLC and metastatic melanoma. In the registrational studies for these indications, the test was not used for patient selection but for a pre-specified retrospective evaluation of the interaction between biomarker expression and clinical benefit from nivolumab single agent (Fig. 5b) [10]. Because of the study design, the advanced disease stage of the patient populations evaluated (i.e., failed standard-of-care), the relative poor NPV of the PD-L1 IHC assay (10–15 % false negative), and the strong association of patient clinical benefit with PD-L1 IHC assay positivity, the use of the test, although not mandated by the FDA for drug prescription in those clinical settings, was approved as a complementary diagnostic to inform prescribers on different risk:benefit from drug administration (i.e., probability of response versus probability of adverse events at the level of the single patient). From a device regulatory point of view, CDx and complementary Dx that have been approved so far in immune-oncology have been classified as Class III devices and have been reviewed through PMA submissions.

### Regulatory considerations for development of predictive biomarkers

As shown in Fig. 1, the biomarker development process can be schematically divided into sequential phases including discovery, research assay optimization, analytical and clinical validation, and commercialization [31]. A premarket submission for approval of the IVD such as a predictive assay should include analytical validity and performance of the IVD in the context of therapeutic use. In addition, if used to guide treatment decisions within a clinical trial, it usually requires an investigational device exemption (IDE) application to the FDA, unless the clinical setting in which the assay is going to be used is considered by the agency “non-significant risk”.

Clinical and analytical requirements for biomarker performance derive from the intended use and should address the following issues:

- Analytical performance demonstrates the ability of the IVD to accurately and reproducibly select patients whose samples contain (or lack) the analyte(s) of interest as a binary variable (e.g., present/absent), a semi-quantitative (e.g., low/medium/high) or a quantitative variable (e.g., level of analyte as related to specified clinical outcome). The core analytical performance of the robust assay must include precision/reproducibility, sensitivity, analytical specificity, limit of detection, linearity and working range, analyte stability and instrumentation performance.
- IVDs utilize a wide range of technologies and platforms to detect and measure DNA, RNA, protein or other substances in the human body. The FDA provides documents intended to guide validation of specific devices to address different platforms including all steps from defining the patient sample type, method of analyte detection, scoring, and proper controls. One such example is a guide for IHC-based tests [32].
- The regulation of novel tests raises new challenges; thus, the FDA is also considering new regulatory approaches to address IVDs based on novel platforms such as genomic tests (e.g., NGS) including algorithm development, computational processing of sequencing data and interpretation of the clinical meaning of individual variables identified [33].
- Algorithms and software used to determine a result of the IVDs application are also reviewed by the FDA. In 2007, the FDA published draft guidance for IVD Multivariate Index Assay (IVDMIA) that describe algorithms derived from complex correlations between large numbers of markers (e.g., index and score) and patient outcome. When software or algorithms are used to generate a single result from the results of multiple tests, these algorithms are considered devices themselves [34].
- The clinical performance of the IVD in selecting patients to receive or avoid a particular therapy or to select a safe and efficacious dose will generally be provided by data from the therapeutic trial(s) indicating that the IVD properly identifies patients for specific treatment choices. This is often dependent on the selection of the appropriate cutoff value that will differentiate patients into the desired outcome classifications (e.g., responders versus non-responders that are above/below a threshold value). A common weakness in exploring candidate biomarkers is that a statistically significant difference in the biomarker levels between patients with good and poor clinical outcomes is identified, but the data overlap and no cutoff is determined. Clinical validation of an IVD in a prospective or retrospective set of samples should use a clinical dataset that is separate from the samples for which the IVD was developed. While prospective studies are ideal for addressing the problem of false associations, alternative techniques using robust retrospective validation or a prospective/retrospective approach may be considered. In many cases, a clinical evaluation of an investigational device must have an IDE before a clinical study is initiated. An IDE approval allows use of an investigational device in a “significant risk” clinical study to collect the data required to support a premarket submission.

### Regulation of biomarkers in the European Union

While the fundamental guiding scientific principles of the regulatory framework for predictive markers such as CDx are similar between the US and EU, significant differences remain. One of the key differences is that the European Medicines Agency (EMA) requires co-development and approval of a CDx at the same time as the drug. However, a harmonization effort is underway to align the key differences between the FDA and EMA guidance on development of CDx. An important proposed change is that CDx will no longer be considered as low risk and subject to self-certification by the manufacturer [35]. According to the new proposal, CDx will be classified as high individual risk such as class III or moderate public health risk (category C) and require conformity assessment by a notified body designated by the EMA [35]. Importantly, both new and existing diagnostics would need to meet these new requirements for safety and performance of diagnostics and on the outcome of the clinical investigation. In cases where clinical investigations are mandatory, these should include randomized control trials in the appropriate target population and well-controlled investigations. Randomized control trials would be considered as the standard appropriate model for all medical diagnostics and sponsors will have to justify any other model chosen.

## Conclusions

Cancer immunotherapies are rapidly changing traditional treatment paradigms and resulting in durable clinical responses in patients with a variety of malignancies. However, the overall number of patients who will respond to these therapies is limited. In addition, there is significant cost as well as potential toxicities that are associated with these therapies that impede their potential clinical impact. Thus, there is a need to develop predictive biomarkers in order to maximize the clinical benefits of this innovative therapy. Although many candidate biomarkers have been described to date, only three assays are FDA-approved (one as a companion and two as a complementary diagnostic) to identify patients who are more likely to benefit from anti-PD-1/PD-L1 therapies. Because of the complexities of both the immune response and of tumor biology, there are unique aspects to the validation process that must be taken into consideration during the planning and implementation phases of biomarker development. In Volume I of this series, we discussed the issues related to the pre-analytical and analytical aspects of biomarker development. Here, in Volume II, we presented aspects of clinical validation and regulatory considerations as they relate to immune biomarker development. Together, this two-volume series discusses the various aspects and provides guidance concerning relevant challenges for the entire biomarker validation process. We believe that the implementation of the recommendations from these guidance documents as well as the other recommended resources will aid in the development and subsequent validation of the most needed, accurate, and precise predictive biomarkers for cancer immunotherapy.

## Additional file

Additional file 1: Publications by the SITC Immune Biomarker Task Force. (DOCX 14.1 kb)

## Abbreviations

CAP: College of American Pathologists
CDRH: Center for Devices and Radiologic Health
CDx: Companion diagnostic
CLIA: Clinical laboratory improvement amendments
CMS: Centers for Medicare and Medicaid Services
EMA: European Medicines Agency
ERCC1: Excision repair cross-complementing 1
FDA: U.S. Food and Drug Administration
FISH: Fluorescence *in situ* hybridization
FPR: False positive rate
IDE: Investigational device exemption
IHC: Immunohistochemistry
IVD: In vitro diagnostic
IVDMIA: IVD Multivariate Index Assay
LDT: Laboratory-developed test
MARVEL: Marker Validation of Erlotinib in Lung Cancer
MIA: Multivariate Index Assay
NCI: National Cancer Institute
NGS: Next generation sequencing
NPV: Negative predictive value
NSCLC: Non-small cell lung cancer
PD-1: Programmed cell death protein 1
PD-L1: Programmed cell death ligand 1
PMA: Premarket approval application
PPV: Positive predictive value
ROC: Receiver operative characteristics
SCNP: Single cell network profiling
TPR: True positive rate
