# Supplementary material for: Validation of biomarkers to predict response to immunotherapy in cancer: Volume II — clinical validation and regulatory considerations
Source: J Immunother Cancer. 2016 Nov 15;4:77. doi: 10.1186/s40425-016-0179-0 (PMC5109653; doi:10.1186/s40425-016-0179-0)
Supplement: Additional file 1: — Publications by the SITC Immune Biomarker Task Force. (DOCX 14.1 kb) [file 40425_2016_179_MOESM1_ESM.docx]

# Additional file 1: Publications by the SITC Immune Biomarkers Task Force

[**Immune monitoring technology primer: immunosequencing**](http://www.immunotherapyofcancer.org/content/3/1/29)

Ilan Kirsch

*Journal for ImmunoTherapy of Cancer* 2015, **3**:29 (25 June 2015)

[**Immune monitoring technology primer: the enzyme-linked immunospot (ELISpot) and Fluorospot assay**](http://www.immunotherapyofcancer.org/content/3/1/30)

Sylvia Janetzki

*Journal for ImmunoTherapy of Cancer* 2015, **3**:30 (21 July 2015)

[**Immune monitoring technology primer: Single Cell Network Profiling (SCNP)**](http://www.immunotherapyofcancer.org/content/3/1/34)

Rachael Hawtin and Alessandra Cesano

*Journal for ImmunoTherapy of Cancer* 2015, **3**:34 (18 August 2015)

[**Immune monitoring technology primer: flow and mass cytometry**](http://jitc.biomedcentral.com/articles/10.1186/s40425-015-0085-x)

Holden T. Maecker and Alexandre Harari

*Journal for ImmunoTherapy of Cancer* 2015, 3:44 (15 September 2015)

[**Immune monitoring technology primer: clinical validation for predictive markers**](http://www.immunotherapyofcancer.org/content/3/1/40)

Kevin K Dobbin

*Journal for ImmunoTherapy of Cancer* 2015, **3**:40 (20 October 2015)

[**Quantitative real-time PCR assisted cell counting (qPACC) for epigenetic-based immune cell quantification in blood and tissue**](http://jitc.biomedcentral.com/articles/10.1186/s40425-015-0087-8)

Thomas Oliver Kleen and Jianda Yuan

*Journal for ImmunoTherapy of Cancer* 2015, 3:46 (17 November 2015)

[**nCounter ^®^ PanCancer Immune Profiling Panel (NanoString Technologies, Inc., Seattle, WA)**](http://www.immunotherapyofcancer.org/content/3/1/42)

Alessandra Cesano

*Journal for ImmunoTherapy of Cancer* 2015, **3**:42 (15 December 2015)

[**Immune Monitoring Technology Primer: protein microarray (‘seromics’)**](http://jitc.biomedcentral.com/articles/10.1186/s40425-016-0106-4)

Jianda Yuan, Ena Wang and Bernard A. Fox *Journal for ImmunoTherapy of Cancer 2016*, 4:2 (19 January 2016)

[**Novel technologies and emerging biomarkers for personalized cancer immunotherapy**](http://jitc.biomedcentral.com/articles/10.1186/s40425-016-0107-3)

Jianda Yuan, Priti S. Hegde, Raphael Clynes, Periklis G. Foukas, Alexandre Harari, Thomas O. Kleen, Pia Kvistborg, Cristina Maccalli, Holden T. Maecker, David B. Page, Harlan Robins, Wenru Song, Edward C. Stack, Ena Wang, Theresa L. Whiteside, Yingdong Zhao, Heinz Zwierzina, Lisa H. Butterfield and Bernard A. Fox

*Journal for ImmunoTherapy of Cancer* 2016, 4:3 (19 January 2016)

[**Multiplexed tissue biomarker imaging**](http://jitc.biomedcentral.com/articles/10.1186/s40425-016-0115-3)

Edward C. Stack, Periklis G. Foukas and Peter P. Lee

*Journal for ImmunoTherapy of Cancer* 2016, 4:9 (16 February 2016)

[**Immune monitoring technology primer: immunoprofiling of antigen-stimulated blood**](http://jitc.biomedcentral.com/articles/10.1186/s40425-016-0122-4)
Laura Rosa Brunet, Samuel LaBrie, Thorsten Hagemann
Journal for ImmunoTherapy of Cancer 2016, **4**:18 (15 March 2016)

[**Immune monitoring technology primer: whole exome sequencing for neoantigen discovery and precision oncology**](http://jitc.biomedcentral.com/articles/10.1186/s40425-016-0126-0)
Pia Kvistborg, Raphael Clynes, Wenru Song, Jianda Yuan
Journal for ImmunoTherapy of Cancer 2016, **4**:22 (19 April 2016)
